# 2D versus 3D laparoscopic total mesorectal excision: a developmental multicentre randomised controlled trial

**DOI:** 10.1007/s00464-018-06630-9

**Published:** 2019-01-17

**Authors:** N. J. Curtis, J. A. Conti, R. Dalton, T. A. Rockall, A. S. Allison, J. B. Ockrim, I. C. Jourdan, J. Torkington, S. Phillips, J. Allison, G. B. Hanna, N. K. Francis

**Affiliations:** 1 Department of Surgery and Cancer, Imperial College London, St Mary’s Hospital, Level 10, Praed Street, London, UK; 2 Department of General Surgery, Yeovil District Hospital NHS Foundation Trust, Higher Kingston, Yeovil, UK; 3 Department of Colorectal Surgery, Portsmouth Hospitals NHS Trust, Southwick Hill Road, Cosham, UK; 4 Academic Surgical Unit, Level C, University of Southampton, University Hospital Southampton, Tremona Road, Southampton, UK; 5 Colorectal Surgery, Royal Surrey County Hospital, Egerton Road, Guildford, UK; 6 The Minimal Access Therapy Training Unit, University of Surrey, Daphne Jackson Road, Guildford, UK; 7 Colorectal Surgery, University Hospital of Wales, Heath Park, Cardiff, UK; 8 Faculty of Science, University of Bath, Wessex House 3.22, Bath, UK

**Keywords:** 3D, Three-dimensional, Laparoscopic, Rectal cancer, Total mesorectal excision, Trial

## Abstract

**Aims:**

The role of laparoscopy in rectal cancer has been questioned. 3D laparoscopic systems are suggested to aid optimal surgical performance but have not been evaluated in advanced procedures. We hypothesised that stereoscopic imaging could improve the performance of laparoscopic total mesorectal excision (TME).

**Methods:**

A multicentre developmental randomised controlled trial comparing 2D and 3D laparoscopic TME was performed (ISRCTN59485808). Trial surgeons were colorectal consultants that had completed their TME proficiency curve and underwent stereoscopic visual testing. Patients requiring elective laparoscopic TME with curative intent were centrally randomised (1:1) to 2D or 3D using Karl Storz IMAGE1 S D3-Link™ and 10-mm TIPCAM®1S 3D passive polarising laparoscopic systems. Outcomes were enacted adverse events as assessed by the observational clinical human reliability analysis technique, intraoperative data, 30-day patient outcomes, histopathological specimen assessment and surgeon cognitive load.

**Results:**

88 patients were included. There were no differences in patient or tumour demographics, surgeon stereopsis, case difficulty, cognitive load, operative time, blood loss or conversion between the trial arms. 1377 intraoperative adverse events were identified (median 18 per case, IQR 14–21, range 2–49) with no differences seen between the 2D and 3D arms (18 (95% CI 17–21) vs. 17 (95% CI 16–19), *p* = 0.437). 3D laparoscopy had non-significantly higher mesorectal fascial plane resections (94 vs. 77%, *p* = 0.059; OR 0.23 (95% CI 0.05–1.16)) but equal lymph node yield and circumferential margin distance and involvement. 30-day morbidity, anastomotic leak, re-operation, length of stay and readmission rates were equal between the 2D and 3D arms.

**Conclusion:**

Feasibility of performing multicentre 3D laparoscopic multicentre trials of specialist performed complex procedures is shown. 3D imaging did not alter the number of intraoperative adverse events; however, a potential improvement in mesorectal specimen quality was observed and should form the focus of future 3D laparoscopic TME trials.

**Electronic supplementary material:**

The online version of this article (10.1007/s00464-018-06630-9) contains supplementary material, which is available to authorized users.

The role of minimal access surgery (MAS) in total mesorectal excision (TME) is hotly contested. Oncological outcomes are closely linked to the technical performance of surgery, specifically through the quality of the TME specimen [1–5]. Medium-term follow-up of multicentre randomised controlled trials (RCTs) suggest that laparoscopic rectal surgery can be performed without oncological compromise [6–8]; however, two recent large RCTs showed that although the majority of laparoscopic cases had acceptable specimens, laparoscopic non-inferiority could not be shown [9, 10]. This topic is highly pertinent as because of perceived short-term patient benefits 68% of UK rectal cancer patients presently receive a laparoscopic operation [7, 11, 12].

The MAS revolution is facilitated by continuous technological development. Advances in laparoscopic platforms include commercially available three-dimensional (3D) HD systems. Initial adoption was hampered by poor image resolution and bulky headgear associated with unacceptable user side effects [13]. Modern refinement of 3D technology has revived surgical interest as contemporary systems have overcome these issues without increasing cognitive load [14–16].

The potential advantages of 3D imaging systems on the performance or outcomes following advanced laparoscopic procedures have not been proved as the available literature predominantly focusses on trainee performance of ex-vivo box trainer tasks with significant methodological concerns raised [14, 16, 17]. Therefore, we designed a development trial with the dual aims of comparing specialist surgical performance of laparoscopic TME surgery using 2D and 3D imaging and to generate evidence to identify and power the appropriate primary endpoint for use in a future definitive TME study.

## Methods

A four-centre, parallel arm (1:1), stage 2b exploration study developmental randomised controlled trial was designed in keeping with the IDEAL recommendations as well as quality assurance in multicentre laparoscopic colorectal trials, 3D laparoscopic studies and CONSORT principles [14, 17–19]. Ethical approval was granted by the UK National Health Service South Central - Berkshire B research ethics committee (16/SC/0118). This trial is registered (ISRCTN59485808).

### Patient eligibility criteria

Study inclusion criteria were biopsy-proven adenocarcinoma of the rectum, ≤ 15 cm from the anal verge, age 18 ≤, provision of written informed consent and the responsible colorectal multi-disciplinary team advised elective laparoscopic TME undertaken with curative intent. Neoadjuvant chemoradiotherapy use remained at the discretion of the responsible clinicians. All patients were required to undergo minimum staging of pelvic MRI, CT chest, abdomen and pelvis, tumour biopsy and full colonic assessment with either optical colonoscopy or CT colonography. Exclusion criteria were known or suspected inflammatory bowel disease, emergency, unplanned or palliative surgery, locally advanced cancers (T4a—TNM 5th edition), refusal or inability to provide informed consent and concurrent or past abdominal or pelvic malignancy. Abdominal-perineal excisions, trans-anal TME and procedures where no anastomosis was planned were also excluded.

### Surgeon eligibility criteria and stereopsis testing

Established experienced minimally invasive rectal cancer centres were approached to participate. All trial surgeons were required to have exceeded previously defined proficiency curve estimates and/or completed the UK LapCo consultant training programme as participant or tutor [20]. Surgeons took the Netherlands organisation for applied scientific research (TNO) stereoscopic visual test (19th edition, Laméris Ootech BV, Utrecht, The Netherlands). Participant stereo acuity was defined as the last correctly reported image with ≤ 120 s of arc considered normal.

### Developmental endpoints and sample size

There was no prior 3D TME research to guide sample size calculations. To assess the impact of stereoscopic imaging on TME performance, the primary endpoint of this study was the total number of enacted intraoperative adverse events per case identified using the observational clinical human reliability analysis (OCHRA) methodology. In previous work, using a combination of open and 2D laparoscopic TME cases, we observed an average of 17 errors (± 7.02 [21]) with differences in specialist performances identified [22]. Using a 5% significant level, a sample size of 62 had 80% power to detect a decrease in error counts to 12. This minimally relevant 30% difference was chosen based on the difference in operative performance of laparoscopic colectomy in the UK LapCo national training programme sign off data as an estimate [22]. Allowing a 15% attrition rate for conversions or loss to follow-up the recruitment target was 72.

### Clinical outcomes

Pre-defined secondary endpoints were operative factors (time, blood loss, stoma creation and conversion—defined as inability to complete the dissection including the vascular ligation and/or requiring an incision larger than that needed for specimen extraction), histopathologically assessed specimen quality (plane of mesorectal excision, lymph node yield, circumferential resection margin and complete excision [2]) and 30-day patient outcomes morbidity (using the Clavien–Dindo classification [23], length of stay and unplanned reattendance or readmission to hospital). As 3D systems have the potential to influence surgeon cognitive load, the NASA-task load index (NASA-TLX) was completed following each case [24]. This widely applied and previously validated surgeon reported system represents the most commonly used measurement method to assess cognition in the operating theatre setting [25, 26].

### Observational clinical human reliability analysis (OCHRA)

To assess whether 3D imaging influenced surgical performance, assessment of the intraoperative period is required to provide detailed analysis of the intervention delivery. The OCHRA technique was adopted in keeping with previous descriptions used for the assessment of specialist performance of laparoscopic colorectal resections and the primary endpoint of a multicentre TME RCT [21, 22, 27]. Briefly, OCHRA involves structured analysis of unedited case video to identify adverse events defined as “something that was not intended by the surgeon, nor desired by a set of rules or an external observer, or that led the task outside acceptable limits” [28]. Events were further categorised by instrument used, external error mode, instrument/dissection or tissue/retraction errors (based upon the perceived principal mechanism for the event) and any resulting consequence used previously reported pre-defined coding lists (Table 2 and Table 4). Errors occur across all task phases not just the pelvis [21, 22, 27], therefore analysis of the entire case was performed. Operative phase of surgery was also captured using a hierarchical task analysis based upon an international consensus [21, 29]. Deviation from this order was not considered as an error. Video review was performed after OCHRA training including blinded analysis of 20 previously recorded 2D laparoscopic TME cases with excellent inter-rater reliability observed (Intraclass correlation co-efficient 0.916).

### Equipment, setup and procedures

All cases were performed using Karl Storz IMAGE1 S D3-Link™ laparoscopic systems with zero or 30° 10-mm TIPCAM®1 SPIES 3D video laparoscopes. Images were displayed on 32-inch LCD HD screens (model EJ-MDA32E-K) and viewed with passive polarising glasses (Panasonic® Europe, Wiesbaden, Germany). To minimise cross-talk and facilitate optimal viewing and ergonomic positioning, precise screen location and viewing distance was at the discretion of each surgical team. All participating surgeons stated that their usual operative plan matched the previously reported international TME standardisation report [29]. To maximise recruitment, generalisability of results and ethical and surgeon acceptability, no constraints on timing of surgery, operative technique, task order, instrument use or any on table decision were made. All perioperative care proceeded as per local site policies.

### Data collection

Video recording utilised the integrated advanced image and data acquisition system (AIDA™, Karl Storz Endoskopy GmBH, Tuttlingen, Germany). Entire cases were recorded unedited in 2D irrespective of randomisation result, deidentified and labelled with a unique study ID as sole identifier. Immediately following case completion, surgeons completed the NASA-TLX instrument and a series of 100-mm visual analogue scales capturing overall case, task and pelvic complexity. Specimen analysis was performed at each site by specialist histopathologists blinded to trial arm and in keeping with the UK Royal College of Pathologists reporting dataset including a three-point ordinal scale for plane of mesorectal dissection. Patients were prospectively followed for 30 days by dedicated research staff independent of the trial. All complications were categorised using the Clavien–Dindo classification [30]. Video files were transferred to the central trial office for analysis using portable hard drives (Canvio Basics, Toshiba Europe, Weybridge, UK). Here, a second coding took place to further ensure blinded analysis.

### Randomisation procedure

To ensure allocation concealment, upon recruitment, patients were randomised centrally to the 2D or 3D arms using a pre-defined computer-generated random number list. Given the sample size, no stratification was undertaken.

### Statistical analyses

The data were analysed using SPSS (v24.0; SPSS Inc, Chicago, IL, USA). All data were explored for normality with the Shapiro–Wilk test and detrended Q–Q plots and compared with parametric or non-parametric tests as appropriate. *t-*test, Mann–Whitney U and Kruskal Wallis testing were used to compare medians from normal and non-normally distributed populations. For categorical data, analysis included the use of cross tabulation, Fisher’s exact test or chi-squared to test association between groups. Effect magnitude was quantified using odds ratio (OR) and 95% confidence intervals. Data are displayed as medians with interquartile ranges (IQR) unless specified. Comparative results are reported as (2D vs.3D) throughout. Analyses are reported as intention to treat except those solely based upon video analysis where the necessity for a complete case recording required a per protocol approach. Statistical significance was defined as *p* < 0.05.

## Results

**Fig. 1:**
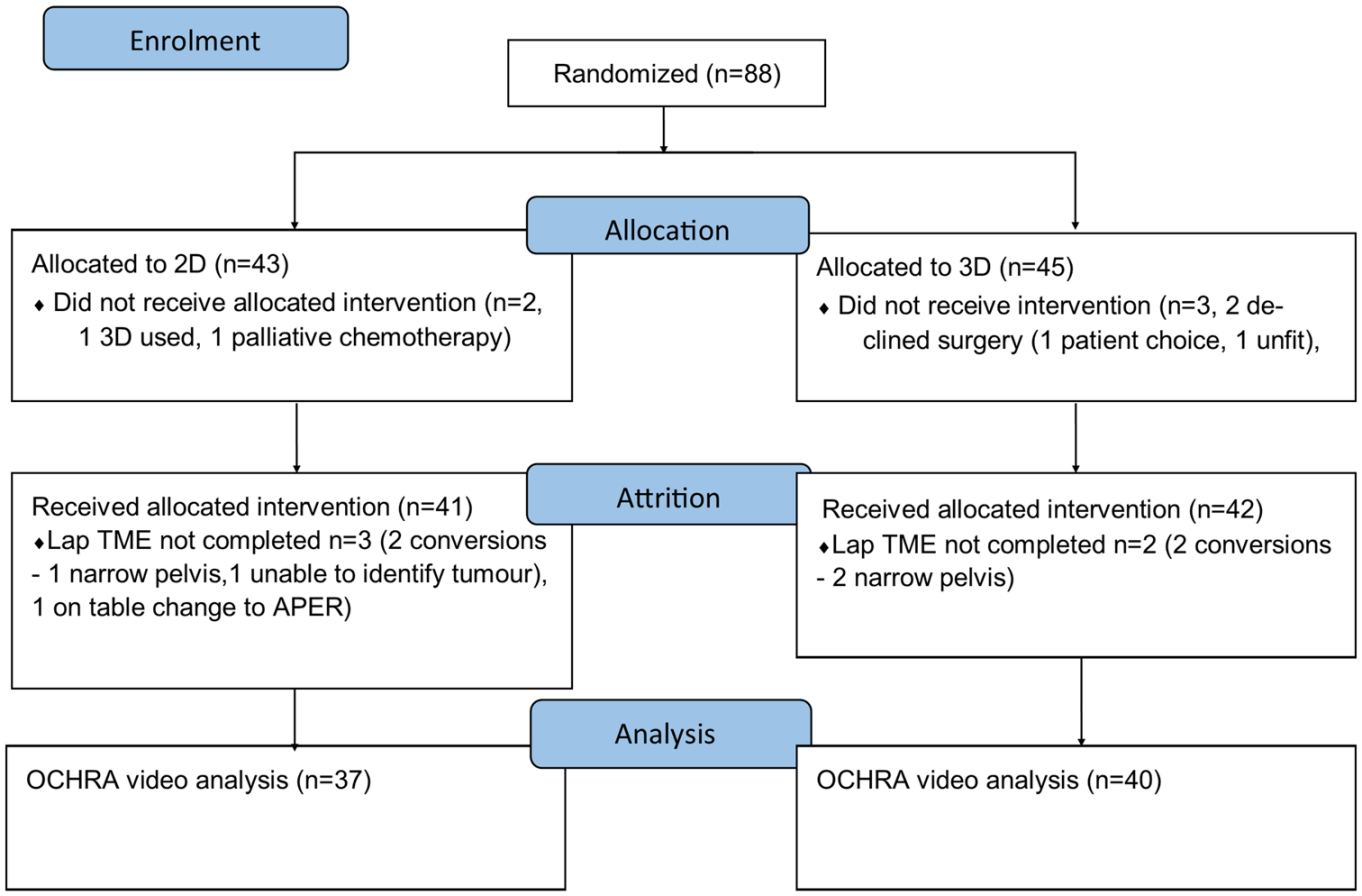
Trial CONSORT diagram. Three patients did not proceed to surgery. Four conversions were seen and with other exclusions 77 videos were available for OCHRA analysis

88 patients from four sites were randomised between June 2016 and March 2018 (Fig. 1). 58% were male. Average age, body mass index and tumour height from the anal verge were 69, 28 and 8.5 cm, respectively. 23% underwent neoadjuvant chemoradiotherapy. All patient and tumour demographics were evenly distributed (Table 1). Nine surgeons participated with no evidence of impaired stereo acuity (range 60–15 s of arc).

**Table 1:** Patient demographics and tumour details

	2D			3D		
	Mean (sd)	Count	Column *N* (%)	Mean (sd)	Count	Column *N* (%)
Age	69 (11)			69 (10)		
Gender						
Females		21	48.8		16	35.6
Males		22	51.2		29	64.4
Body mass index	29 (5)			27 (4)		
Previous abdominal or pelvic surgery						
No		29	67.4		33	73.3
Yes		14	32.6		12	26.7
American society of anaesthesiologists score						
I		4	9.3		2	4.4
II		24	55.8		28	62.2
III		11	25.6		14	31.1
IV		3	7		0	0.0
Unknown		1	2.3		1	2.2
Neoadjuvant use						
None		32	74.4		36	80.0
Short course radiotherapy		1	2.3		0	0.0
Long course chemoradiotherapy		10	23.3		9	20.0
Tumour height (cm)	8.5 (3)			8.4 (3.1)		
Tumour height from anal verge						
Upper (10.1–15 cm)		10	23.3		14	31.1
Mid (6.1–10 cm)		23	53.5		18	40
Lower (≤ 6 cm)		10	23.3		13	28.9
Predominant tumour location						
Anterior		14	32.6		11	24.4
Posterior		9	20.9		7	15.6
Left lateral		8	18.6		7	15.6
Right lateral		2	4.7		7	15.6
Circumferential		9	20.9		11	24.4
Unknown		1	2.3		2	4.4

All key patient, tumour and neoadjuvant therapy factors were equally distributed between trial arms. Tumours were predominantly mid-rectal but included equal numbers of upper and lower rectal cancers

### Operative data and surgeon reported case complexity

No differences were seen in surgeon reported overall case complexity (28 mm (IQR 18–43) vs. 31 mm (19–63), *p* = 0.399), any surgical phase or pelvic quadrants between the trial arms (Table 2). No differences in surgical time (278 (95% CI 270–360) vs. 270 min (235–335), *p* = 0.34), blood loss (60 vs. 90 ml, *p* = 0.618), conversion (2 (4.9%) vs. 2 (4.8%), *p* = 0.981), defunctioning ileostomy creation (89% vs. 85%, *p* = 0.587) or anastomosis height (3 vs. 3 cm, *p* = 0.829) were seen.

**Table 2:** Surgeon reported case difficulty

	2D	3D	*p*
	Median	Median	
Overall case complexity	28	31	0.399
Access to abdomen	14	13	0.784
Splenic Flexure mobilisation	21	18	0.127
IMA pedicle dissection and division	22	20	0.871
Access to pelvis	16	18	0.511
Identification of autonomic nerves	24	22	0.54
Division of rectum	19	20	0.919
Anastomosis	22	17	0.181
Anterior TME			
Anterior TME difficulty	30	25	0.78
Oedema	5	6	0.483
Fibrosis	8	8	0.327
Bleeding	6	8	0.4
Surgical planes	14	13	0.838
Left lateral TME			
Left TME difficulty	19	22	0.705
Oedema	7	9	0.676
Fibrosis	7	10	0.363
Bleeding	9	10	0.86
Surgical planes	14	16	0.68
Right lateral TME			
Right TME difficulty	25	30	0.29
Oedema	7	7	0.616
Fibrosis	10	14	0.316
Bleeding	9	12	0.504
Surgical planes	20	20	0.38
Posterior TME			
Posterior TME difficulty	20	18	0.603
Oedema	7	6	0.524
Fibrosis	8	7	0.593
Bleeding	8	8	0.941
Surgical planes	16	13	0.383

100-mm visual analogue scales with 0 representing the easiest possible case were used. All figures are medians. No difference in any measure is seen between the trial arms so the Bonferroni correction was not applied. Overall the scores are relatively low for a complex procedure

### Short-term patient outcomes

A total of 110 morbidity events from 52 patients were recorded in the first 30 post-operative days (any morbidity 61.2%, median 1 per patient, IQR 0–2, range 0–5, Table 3) with no difference between trial arms (59.5% vs. 62.7%, odds ratio 1.2 (95% CI 0.5–2.9), *p* = 0.834) or Clavien–Dindo classification (*p* = 0.899). Anastomotic leak rate (overall 5.9%, 4.8% vs. 7%, *p* = 0.666) and re-operation rate (7.1% vs. 4.7%, *p* = 0.666) were comparable between the arms. Non-significant differences in length of hospital stay (9 (IQR 6–18) vs. 7 (5–15) days, *p* = 0.203) and re-admissions were observed (11.9% vs. 25.6%, *p* = 0.109).

**Table 3:** 30-day morbidity events with Clavien–Dindo classification [30]

Trial Arm Number of cases	2D 42				3D 43			
Clavien–Dindo classification	I	II	III	IV	I	II	III	IV
Ileus	5	4			5	3		
Acute kidney injury	2	3			4	2		
Urinary retention	3				4	1		
Wound infection		5				1	1	
Sepsis		4				3		
Abdominal or pelvic collection		2	2			2	1	
High output stoma	1	1			1	3		
Urinary tract infection		4			1	1		
Atrial fibrillation, flutter or supraventricular tachycardia		3			1			1
Anastomotic leak			2**				3**	
Anaemia						2		
Hypertension					1	1		
Nausea/vomiting	1		1					
Stoma prolapse					2			
Pneumonia		1				1		
Splenic haematoma	1				1			
Allergic reaction					1			
Chest pain					1			
Diabetic ketoacidosis		1						
Duodenal ulcer bleed							1	
High output drain	1							
Hypocalcaemia						1		
Hypotension								1
Ischaemic optic neuropathy	1							
Neuropraxia					1			
Neutropenia							1	
Pancreatitis				1				
Rectal bleeding					1			
Retrograde ejaculation	1							
Small bowel obstruction			1*					
Stomal bleeding	1							
Stomatitis		2						
Vasovagal collapse	1							
Wound bleeding	1							
Sum	19	30	6	1	24	21	7	2
Total	56				54			

Number and nature were evenly distributed between trial arms (*p* = 0.899) with no differences seen in anastomotic leak or reo-peration rates. 40% of 2D patients and 37% of 3D patients recovered without developing any morbidity event. Asterisk denotes a re-operation took place for this indication

### OCHRA analysis

**Fig. 2:**
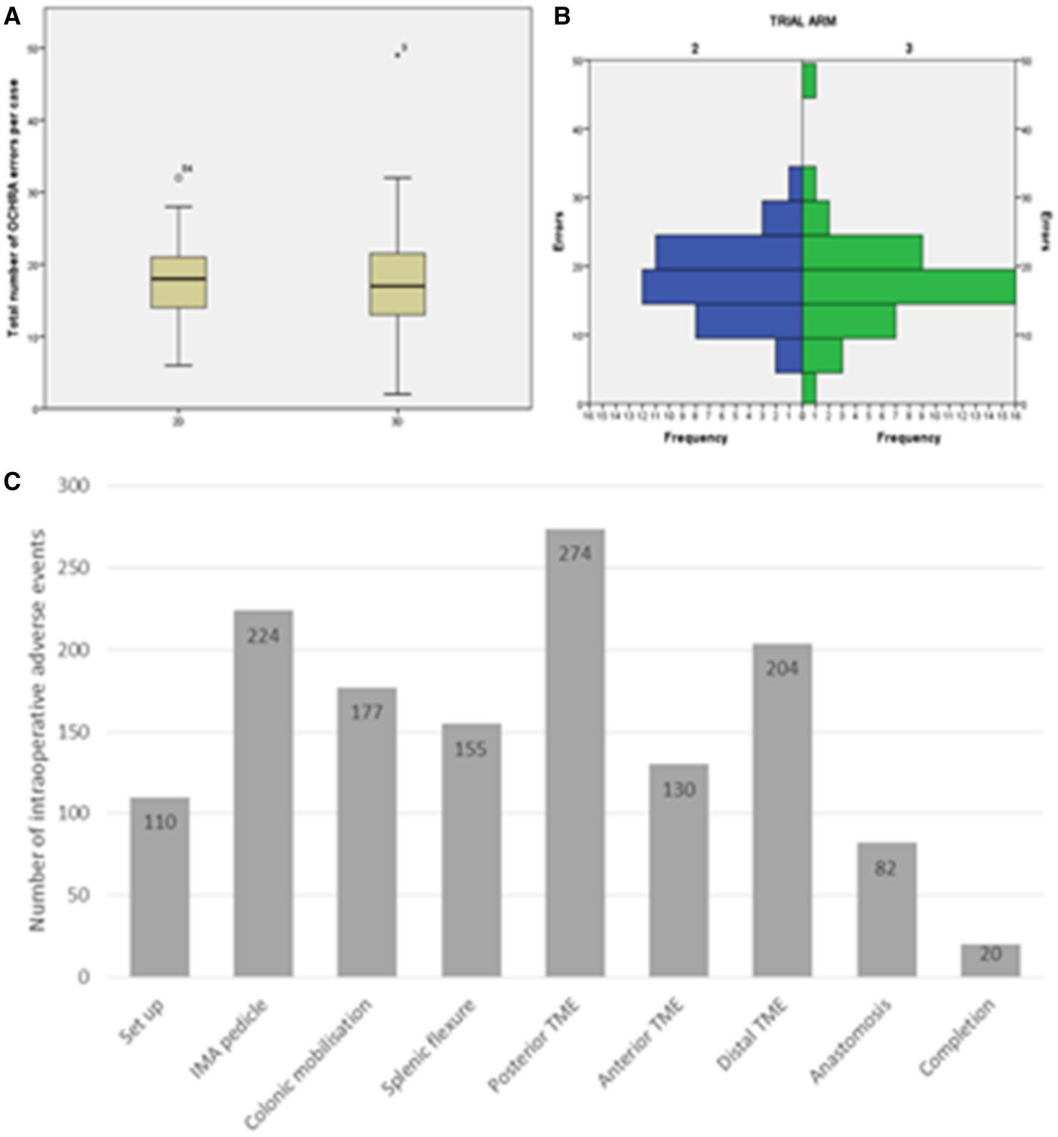
**A–C** Intraoperative error data. **A** Box and whisker plot, **B** histogram, **C** errors per operative phase. No differences in the distributions are seen. Errors were seen to take place across all phases of the operation justifying the approach to review entire cases. Studying pelvic performance alone would have missed 50% of identified adverse events

77 cases were analysed comprising 380 h of surgery. A total of 1377 intraoperative errors were identified (median 18 per case, IQR 14–21, range 2–49). No differences were seen between the 2D and 3D arms (18 (IQR 14–21) vs. 17 (IQR 13–22), *p* = 0.437). OCHRA categorical data are displayed in Fig. 2A–C and Table 4. Apart from a reduction in overshoot errors in 3D surgery (64 vs. 48, *p* = 0.05), no differences are seen in the data. Errors took place across all operative phases with 689 (50%, Fig. 2) taking place during pelvic tasks; however, no difference between the trial arms was seen (total 322 vs. 367, median 8 per case (6–12) vs. 8 (6–11), *p* = 0.854) or by pelvic location (Supplementary Table 1 + Supplementary Fig. 1).

**Table 4:** OCHRA categorical data

	2D	3D	
	Sum	Sum	*p*
Number of laparoscopic TME cases	37	40	
Errors—dissection/instrument use			
Poor visualisation of tip	45	46	0.415
Overshoot of movement	64	48	0.05
Instrument applied with too little distance to structure	59	53	0.428
Inappropriate use of diathermy/energy source	15	16	0.995
Incorrect amount of energy applied	36	55	0.426
Dissection performed in wrong direction	40	28	0.086
Diathermy/dissection in wrong tissue plane	136	145	0.801
Use of inappropriate energy to dissect	27	19	0.415
Cutting without lifting tissues from underlying structures	18	13	0.404
Errors—retraction/tissue handling errors			
Avulsion of tissue	27	33	0.837
Too much blunt force applied to tissue	73	88	0.340
Traction applied with too much tension	47	65	0.306
Traction applied with too little tension	23	17	0.426
Traction applied in wrong direction	16	14	0.911
Inappropriate handling of tumour	3	3	0.921
Inappropriate grasping/blunt handling of structure	42	51	0.541
Use of inappropriate instrument to retract	7	13	0.288
Consequences			
Bleeding (ooze)	229	233	0.558
Bleeding (significant/pulsatile)	25	44	0.365
Mesorectal injury—breech of fascia only	37	47	0.324
Mesorectal injury—into mesorectal fat	29	51	0.154
Mesorectal injury—exposing rectal adventitia	10	6	0.402
Mesorectal injury—into rectal musculature	1	1	0.956
Rectal perforation	5	1	0.074
Diathermy burn to viscus	31	33	0.553
Sharp injury to viscus	4	6	0.38
Blunt bowel injury	15	15	0.821
Perforating bowel injury	1	2	0.605
Diathermy burn to other structure	11	11	0.599
Sharp injury to other structure	2	2	0.937
Risk of pelvic nerve injury	19	17	0.713
Injury to pelvic nerves	20	15	0.54
Injury to pelvic fascia	19	12	0.253
Injury to ureter	0	0	1
Risk of injury to other structure	19	26	0.561
Injury to other structure	19	22	0.957
Delay to progress of operation	10	13	0.36
Oncological compromise of operation	3	7	0.337
External error mode			
Step not done	24	23	0.394
Step partially completed	30	36	0.838
Step repeated	21	19	0.48
Second additional step	14	10	0.892
Second step performed instead	0	3	0.171
Step out of sequence	3	5	0.531
Step done with too much force, speed, depth, distance, time or rotation	237	263	0.841
Step done with too little force, speed, depth, distance, time or rotation	58	61	0.454
Step done in wrong orientation, direction or point in space	167	170	0.603
Step done on/with wrong object	112	111	0.472
Instrument			
Hook diathermy	138	123	0.528
Finger switch diathermy	1	0	0.298
Ultrasonic dissection	249	287	0.846
Johann grasper	208	207	0.347
Fine grasper	3	6	0.608
Swab	4	3	0.895
Suction	6	11	0.853
Scissors	4	13	0.136
Stapler	24	15	0.104
Bowel clamp	0	2	0.336
Clip applicator	6	10	0.191
Retractor	1	1	0.956
Other instruments	21	20	0.348
Hierarchical surgical task phase			
Setup	42	68	0.317
Vascular pedicle	121	103	0.174
Colonic mobilisation	90	87	0.406
Splenic flexure	85	70	0.329
Posterior TME	122	152	0.374
Anterior TME	60	70	0.766
Distal TME	94	110	0.922
Resection and anastomosis	47	35	0.142
Completion, stoma and closure	8	12	0.507

All figures represent the sum of observed events. The number and nature of observed adverse events are in keeping with those expected for expert performed laparoscopic total mesorectal surgery with serious events infrequently seen. The only identified difference is a reduction of overshoot errors in the 3D cases as could result from an increase in depth perception provided by stereopsis

### Surgeon cognitive load

**Fig. 3:**
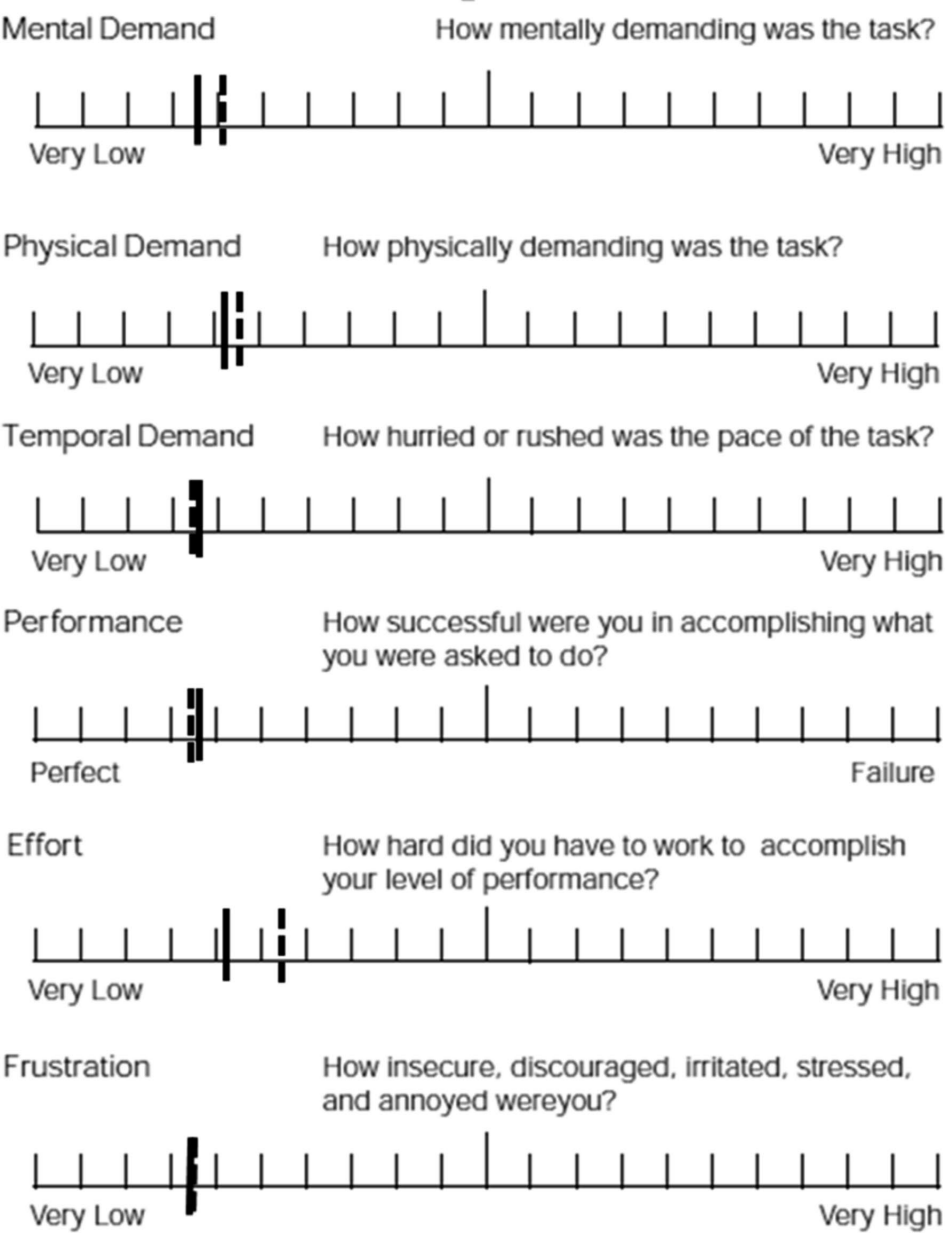
NASA-TLX with medians displayed (2D—dashed line, 3D—solid line). Overall low demands were reported in both arms and were not influenced by the use 2D or 3D imaging (*p* = 0.59, 0.825, 0.64, 0.942, 0.270 and 0.286, respectively)

Surgeons reported low demands across all six domains of the NASA-TLX with no statistical or clinically relevant differences seen between the trial arms (Fig. 3).

### Specimen analysis

**Fig. 4:**
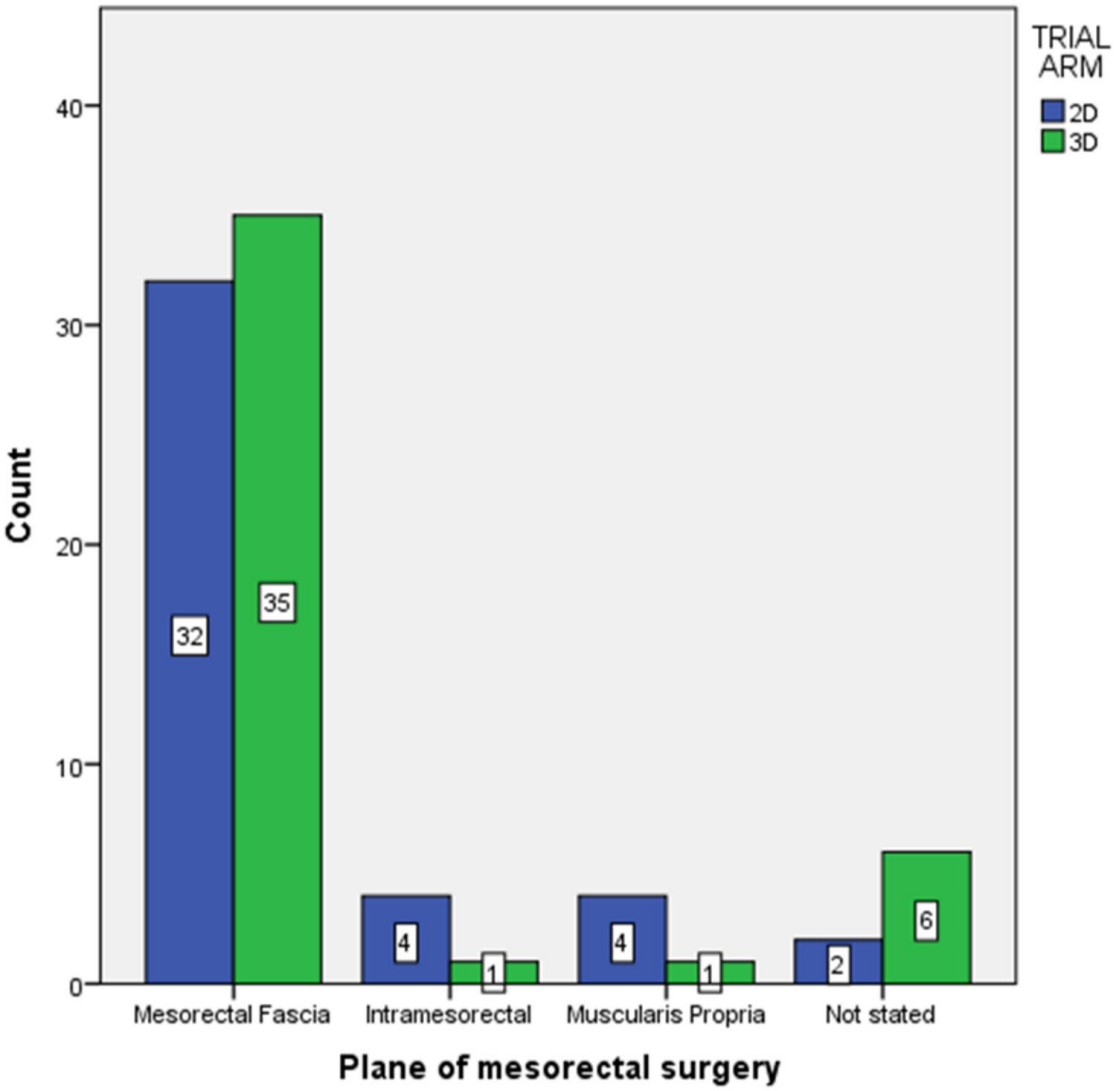
Histopathological assessment of the mesorectal surgical plane. Despite inclusion in the UK Royal College of Pathologists colorectal cancer dataset was not given in eight (9.4%) reports. When these are excluded a clinically significant increase in mesorectal fascial plane surgery is seen (87% overall, 77% vs. 94%, OR 0.23 (95% CI 0.05–1.16), *p* = 0.059)

Pathologically assessed tumour stages, relationship to the peritoneal reflection, lymph node yield and circumferential resection margins were equal between 2D and 3D surgery (Table 5). A single R1 resection was observed in each arm (*p* = 0.987). Intention-to-treat analysis showed no difference in mesorectal fascial plane surgery (76% vs. 81%, OR 0.73 (95% CI 0.26–2.08), *p* = 0.163). However, the plane was not reported in eight cases (9.4%) predominantly from 3D patients. When these were excluded, 3D laparoscopy produced clinically but not statistically significant higher rates of mesorectal plane excisions (77% vs. 94%, OR 0.23 (95% CI 0.05–1.16), *p* = 0.059, Fig. 4).

**Table 5:** Histopathology data

	2D		3D		*p*
	Count	Column *N* (%)	Count	Column *N* (%)	
Tumour stage					
PCR	0	0.0	2 (22% PCR rate)	4.7	0.658
1	15	35.7	13	30.2	
2	13	31.0	15	34.9	
3	13	31.0	12	27.9	
4	1	2.3	1	2.3	
pT					
PCR	0	0.0	2	4.7	0.497
1	4	9.5	6	14.0	
2	18	42.9	9	20.9	
3	18	42.9	22	51.2	
4	2	4.8	4	9.3	
pN					
0	28	66.7	31	72.1	0.687
1	9	21.4	6	14.0	
2	5	11.9	6	14.0	
pM					
0	41	97.6	42	97.7	1
1	1	2.4	1	2.3	
Relationship to peritoneal reflection					
Above	22	52.4	18	41.9	0.188
Astride	8	19.0	6	14.0	
Below	12	28.6	19	44.2	
Circumfrential resection margin (mm, median, IQR)	17.0 (10–25)		11.0 (6–18)		0.088
Lymph node yield total (median, IQR)	19 (15–27)		19 (14–26)		0.912
Plane of mesorectal excision					
Mesorectal	32	76.2	35	81.4	0.163
Intramesorectal	4	9.5	1	2.3	
Muscularis propria	4	9.5	1	2.3	
Not reported	2	4.8	6	14	
R status					
0	41	97.6	42	97.7	0.987
1	1 (CRM 0.8 mm)	2.4	1 (distal margin < 1 mm)	2.3	

No differences are observed between the arms although a clinically relevant but non-significant increase in mesorectal plane surgery is seen in the 3D arm. PCR—Pathological complete response to neoadjuvant chemotherapy

## Discussion

With the present debate on the role of MAS in rectal cancer surgery, appraisal of novel technology that may positively impact on outcomes is required. There has been an uptake in 3D laparoscopy in clinical settings despite little evidence to support its use. Since there was no prior research, and as advocated by the IDEAL collaboration on surgical innovation, it was important to perform a developmental study in order to assist the design a future definitive RCT [18]. Feasibility of the methodology and multicentre recruitment was also needed given the time and resource implications of major trials. Here, we incorporated all methodological recommendations for multicentre laparoscopic colorectal RCTs and 3D studies [14, 17, 19] and report the first TME trial using 3D laparoscopy.

Assisted by video capture technology integrated in most MAS platforms, we deliberately studied the frequently overlooked intraoperative period as it was felt this is where any impact of imaging technology was most likely to be seen. It was hoped this could provide new insights into trial findings and identify areas for targeted improvements. Using the validated, structured OCHRA technique which we previously successfully applied to the assessment of intraoperative specialist performance and as the primary endpoint of a multicentre TME RCT, provision of stereoscopic imaging did not alter the number of enacted error events. Although a margin of 30% was selected, the observed difference was nominal supporting our approach to perform this preliminary trial. Video review is hindered by its time-intensive nature and importantly did not link operative performance to specimen results. Therefore, its relevance is questionable and appears redundant in future TME studies.

Optimal oncological outcomes are obtained through achieving a complete TME resection including clear circumferential margins and mesorectal fascial plane surgery [1, 2, 4, 5, 31–33]. Our main finding was the potential improvement in TME specimen quality following 3D laparoscopy. No other differences were observed across any other outcome. 94% of 3D TME specimens were assessed as mesorectal fascial plane representing a clinically, but borderline statistically, significant improvement over 2D surgery. This figure exceeds the results reported by major laparoscopic rectal cancer trials including their open and robotic arms [6, 9, 10, 34]. Resection in the mesorectal fascial plane is associated with reduced local and distant recurrence and improvements in disease-free and overall survival. This result and the very low CRM involvement rate can be expected to lead to low rates of recurrence and together with the acceptable conversion, leak and re-operation rate support the ongoing use of laparoscopy by specialist surgeons. It should be noted that reflecting our exclusion of abdominal-perineal resections the average tumour height was slightly higher than the major trials and lower neoadjuvant use was seen in keeping with UK guidelines and practice.

Across all other pre-defined endpoints, equivalence between the 2D and 3D trials arms was seen. The equal operative, cognitive load and patient outcome data suggest specialist performance was not altered by the imaging technology used. It is possible their experience has overcome the lack of depth perception inherit to 2D laparoscopy. No meaningful surgeon side effects were encountered and no deterioration in cognitive load was seen suggesting contemporary 3D platforms have indeed overcome past deficiencies [13, 16]. Our results are strengthened by the use of centralised randomisation with allocation concealment, standardised equipment across all centres, stereopsis testing, blinded video assessment and independent histopathology and morbidity data collection.

Given the current literature concerns regarding laparoscopic TME specimen quality, our findings warrant further exploration. Mesorectal plane of excision should be adopted as the primary endpoint for a future larger multicentre RCT and would be additionally strengthened by the use of centralised, protocol-led specimen review. Our study design was agreeable to patients, surgeons and theatre teams resulting in acceptable recruitment with low attrition which should be reproducible across additional sites. Should a definitive study confirm our findings this would represent an easily implemented and generalisable route to quality improvement whilst delivering the short-term recovery benefits presented by MAS [7, 12]. Outside this endpoint, the equivalence of all other data does not support undertaking larger trials. To provide homogeneity, we excluded abdominal-perineal and trans-anal TME excisions. Although the need for a complete specimen is unaltered, variation in perineal and low rectal technique could have directly influenced histopathology results. The health economics of 3D laparoscopy have not been sufficiently reported to date although a recent health technology assessment suggested the additional cost per patient for 3D systems in general surgery could be as low as €1.67 [15]. Our data suggest no meaningful secondary impact on healthcare resources could be expected.

Surgical intervention research presents specific challenges but the need for evidence-based practice remains including in the use of theatre technologies [18]. It remains surprising that surgical technology undergoes intensive development and testing to obtain licencing but clinical research assessment is not mandatory. This is in direct contradiction to the extensive regulatory requirements for other healthcare interventions such as pharmaceuticals. The few randomised clinical 3D studies have also shown equivalent results going back over 20 years [35]. Randomisation removes many of the inherent biases that can unduly influence comparable studies. Our trial surgeons subjectively praised 3D systems and were surprised when data were unblinded in a similar fashion to other colorectal MAS technology trials [34, 36]. The majority of 3D laparoscopy studies have used box trainers and laparoscopically naïve participants limiting the applicability to OR performance [16, 17].

This study should be considered in view of its limitations. In nearly 10% of cases, no mesorectal plane assessment was given despite being a core requirement for TME histology reporting. These data may have influenced our conclusions, but early identification of this issue shows the strength of undertaking preliminary studies and will improve future RCT design. Although we successfully met our aims, as a developmental study with a modest sample size, firm conclusions should not be drawn. We complied with the CONSORT criteria however laparoscopic case selection bias cannot be fully excluded as pre-operative decision making and open TME surgery performed at each centre during the study timeframe were not captured. Although cognitive load was measured, case video does not capture human factors including team experience, interaction and distraction that could influence surgeon performance or the extracorporeal operative tasks. The 500 h of video analysis undertaken here highlights the limited applicability to routine clinical practice. Finally, the results obtained reflect the expertise of the participating surgeons and their centres and cannot be assumed to be applicable to trainees or inexperienced laparoscopic TME surgeons.

## Conclusion

Feasibility of performing multicentre 3D laparoscopic multicentre trials of specialist performed complex procedures is shown. 3D imaging did not alter the number of intraoperative adverse events; however, a potential improvement in mesorectal specimen quality was observed and should form the focus of future 3D laparoscopic TME trials.

## Electronic supplementary material

Below is the link to the electronic supplementary material.

Supplementary material 1 (DOCX 14 KB)

Supplementary material 2 (TIF 1608 KB)
