# Supplementary material for: 2D versus 3D laparoscopic total mesorectal excision: a developmental multicentre randomised controlled trial
Source: Surg Endosc. 2019 Jan 17;33(10):3370–83. doi: 10.1007/s00464-018-06630-9 (PMC6722156; doi:10.1007/s00464-018-06630-9)
Supplement: Supplementary file 1 — Supplementary material 1 (DOCX 14 KB) [file 464_2018_6630_MOESM1_ESM.docx]

**Supplementary table 1**

| Pelvic error location | 2D | |  | 3D |  |  |
| --- | --- | --- | --- | --- | --- | --- |
|  | Sum | | % | Sum | % | p |
| Right anterior | | 28 | 15.2% | 31 | 17.4% |  |
| Right lateral | | 14 | 9% | 13 | 13% |  |
| Right posterior | | 21 | 9.9% | 18 | 10.1% |  |
| Posterior | | 22 | 28.9% | 20 | 22.3% |  |
| Left posterior | | 16 | 6.2% | 18 | 5.7% |  |
| Left lateral | | 24 | 10.2% | 22 | 13.6% |  |
| Left anterior | | 19 | 20.5% | 20 | 17.7% |  |
| Total | | 322 |  | 367 |  | 0.854 |
