# Supplementary figures and images for: 2D versus 3D laparoscopic total mesorectal excision: a developmental multicentre randomised controlled trial
Source: Surg Endosc. 2019 Jan 17;33(10):3370–83. doi: 10.1007/s00464-018-06630-9 (PMC6722156; doi:10.1007/s00464-018-06630-9)

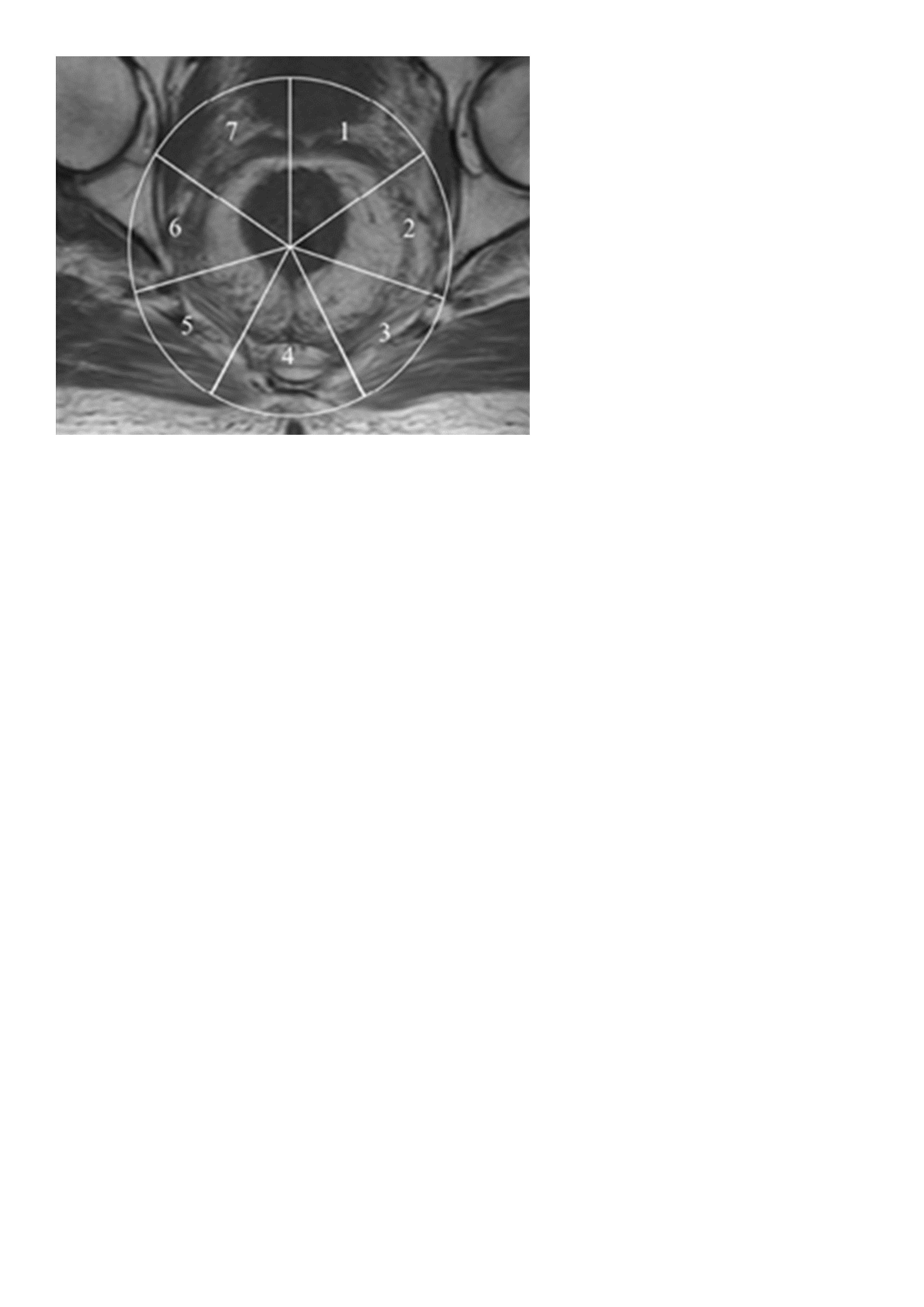

Supplement: Supplementary file 2 — Supplementary material 2 (TIF 1608 KB) [file 464_2018_6630_MOESM2_ESM.tif]
